# Beyond Recycling Antibodies: Crovalimab’s Molecular Design Enables Four-Weekly Subcutaneous Injections for PNH Treatment

**DOI:** 10.3390/ijms252111679

**Published:** 2024-10-30

**Authors:** Zenjiro Sampei, Kenta Haraya, Siok Wan Gan, Masaru Muraoka, Akira Hayasaka, Taku Fukuzawa, Meiri Shida-Kawazoe, Yoshinori Tsuboi, Akihiko Gotoh, Naoshi Obara, Yasutaka Ueda

**Affiliations:** 1 Chugai Pharmaceutical Co., Ltd., Yokohama 244-8602, Japan; 2 Chugai Pharmabody Research Pte., Ltd., Singapore 138623, Singapore; 3 Department of Hematology, Tokyo Medical University, Tokyo 160-0023, Japan; 4 Department of Hematology, Faculty of Medicine, University of Tsukuba, Ibaraki 305-8576, Japan; 5 Department of Hematology and Oncology, Graduate School of Medicine, Faculty of Medicine, Osaka University, Osaka 565-0871, Japan

**Keywords:** paroxysmal nocturnal hemoglobinuria, crovalimab (PiaSky^®^), anti-C5 antibody, subcutaneous injection, viscosity, charge engineering, pH-dependent antigen binding, recycling antibody, eculizumab, ravulizumab

## Abstract

The advent of recycling antibodies, leveraging pH-dependent antigen binding and optimized FcRn interaction, has advanced the field of antibody therapies, enabling extended durability and reduced dosages. Eculizumab (Soliris^®^) demonstrated the efficacy of C5 inhibitors for paroxysmal nocturnal hemoglobinuria (PNH), while its derivative, ravulizumab (Ultomiris^®^), recognized as a recycling antibody, extended the dosing intervals. However, limitations including intravenous administration and inefficacy in patients with the R885H single-nucleotide polymorphism (SNP) in C5 could necessitate alternative solutions. Crovalimab (PiaSky^®^), a next-generation recycling antibody, overcomes these challenges with innovative charge engineering, achieving the enhanced cellular uptake of C5–crovalimab complexes and targeting a unique C5 epitope, allowing for efficacy regardless of the R885H SNP. This study highlights crovalimab’s distinctive molecular features, showing its eliminated binding to Fcγ receptors and C1q, alongside its optimized antigen binding characteristics. The impact of charge engineering was reconfirmed in mice, demonstrating faster C5 clearance than recycling antibodies. Notably, in the maintenance dosing regimen, crovalimab neutralizes approximately seven C5 molecules per antibody on average. Furthermore, its design also reduces the viscosity to facilitate high-concentration formulations suitable for subcutaneous delivery. Consequently, crovalimab offers a four-weekly subcutaneous injection regimen for PNH, marking a substantial improvement in treatment convenience and potentially transforming patients’ quality of life.

## 1. Introduction

Paroxysmal nocturnal hemoglobinuria (PNH) is a rare, acquired, hematologic disorder [1,2], with an estimated prevalence of 10.4 to 38.1 per million individuals and incidence of 0.08 to 0.57 per 100,000 person-years [3,4,5]. PNH is caused by somatic mutations in the phosphatidylinositol glycan class A (*PIGA*) gene [6,7] that lead to a deficiency of glycosylphosphatidylinositol-anchored proteins, including CD55 and CD59 [8,9,10]. The loss of CD55 and CD59 renders PNH erythrocytes vulnerable to complement-mediated intravascular hemolysis [11,12], which results in clinical manifestations of PNH, including hemolytic anemia, thrombosis, cytopenias, and renal insufficiency [13]. Monoclonal antibodies that target complement C5 and inhibit the activation of terminal complement activity (C5 inhibitors) are the current standard of care for PNH, where available [14,15], and have been used in clinical and real-world settings for over 20 years [16].

Eculizumab (Soliris^®^, Alexion Pharmaceuticals, Inc., Boston, MA, USA), a first-in-class monoclonal antibody C5 inhibitor, was approved for the treatment of PNH based on its efficacy in two phase III clinical trials (TRIUMPH and SHEPHERD) [17,18]. Eculizumab requires intravenous (IV) dosing every 2 weeks (Q2W) and is effective in reducing intravascular hemolysis, decreasing the levels of lactate dehydrogenase (LDH), and lowering the need for blood transfusions [17,18,19]. However, the need for IV infusions Q2W, typically in a supervised healthcare setting, could be considered a treatment burden for some patients [11,20].

Ravulizumab (Ultomiris^®^, Alexion Pharmaceuticals, Inc., Boston, MA, USA) is an engineered derivative of eculizumab that binds to the same epitope on C5 as eculizumab. It was created through histidine substitutions [21], allowing ravulizumab to bind to C5 in a pH-dependent manner. Furthermore, ravulizumab was engineered to increase the efficiency of the neonatal Fc receptor (FcRn)-mediated salvage process and prolong the terminal plasma half-life [21]. This allows for extended IV dosing intervals (every 8 weeks [Q8W]) compared with eculizumab [21]. In two phase III trials in C5 inhibitor-naive (Study 301) and C5 inhibitor-experienced (Study 302) patients, ravulizumab demonstrated non-inferior efficacy to eculizumab, providing equivalent LDH normalization, transfusion avoidance, and the control of breakthrough hemolysis [22,23,24]. These results led to the first approvals of ravulizumab in 2018. A subcutaneous (SC) formulation of ravulizumab, administered weekly, was also approved in a few countries, but it is not currently available for use [25,26].

Although eculizumab and ravulizumab provide considerable clinical benefits and have transformed the landscape of PNH treatment, there remain some patient needs that are yet to be fully addressed [11,14,15]. The necessity for regular IV infusions of these medications, typically administered in a supervised healthcare setting (with home-based therapy available in some countries), could be viewed as a considerable challenge, potentially impacting patients’ quality of life [11,20,27,28]. Additionally, it has been reported that patients with the R885H single-nucleotide polymorphism (SNP) in C5 (c.2654GàA [Arg885His]) face challenges regarding the therapeutic efficacy of eculizumab and ravulizumab, due to the insufficient binding affinity between the antibodies and the R885H C5 [22,29].

The need for the IV administration of eculizumab and ravulizumab primarily stems from the high dosage of antibodies required to achieve therapeutic efficacy. This is due to the large number of C5 molecules to be neutralized, which is further elevated by the C5 accumulation in plasma. In general, administering antibody therapeutics targeting soluble antigens can lead to the formation of immune complexes between the administered antibodies and the soluble antigens, which slows the clearance of these antigens, resulting in antigen accumulation in the bloodstream. As shown in Figure S1, when the clearance rate of the soluble antigen from the plasma prior to antibody administration (CL[Ag]) equals the clearance rate of these immune complexes from the plasma (CL[IC]), antigen accumulation in the plasma does not occur. However, as the ratio of CL(Ag)/CL(IC) increases—indicating a reduction in antigen clearance due to antibody binding—there is a notable rise in the total antigen concentration, alongside an increase in the concentration of free, unbound antigen. Therefore, it is important to modulate the clearance of immune complexes from the plasma, i.e., accelerate the cellular uptake of these complexes to enhance the efficiency of antibody therapeutics by minimizing antigen accumulation and, consequently, reduce the dosage required for complete antigen neutralization.

The Recycling Antibody^®^ (Chugai Pharmaceutical Co., Ltd., Tokyo, Japan), created using Chugai’s proprietary pH-dependent antigen-binding technology and optimized FcRn interaction [30], addresses these issues. Its pH-dependent antigen binding property allows antibodies to neutralize antigens in the neutral pH of plasma and release them under the acidic pH of endosomes after cellular uptake. This facilitates the lysosomal degradation of antigens and enhances the antigen clearance, while antigen-free antibodies are efficiently recycled back to the plasma with its optimized FcRn interaction. Through repeating this cycle, a single recycling antibody molecule can neutralize antigens multiple times, extending the durability and reducing the required dosage. Satralizumab (Enspryng^®^, Chugai Pharmaceutical Co., Ltd., Tokyo, Japan) is the first antibody created by applying our proprietary recycling antibody technology [30]. Ravulizumab is also recognized as a recycling antibody due to its pH-dependent C5 binding and enhanced FcRn binding properties [21].

Crovalimab (PiaSky^®^, Chugai Pharmaceutical Co., Ltd., Tokyo, Japan; previously RO7112689 or SKY59) is a next-generation recycling antibody that employs Chugai’s proprietary recycling antibody technology and novel surface-charge engineering. The engineered surface charge is a key difference between crovalimab and ravulizumab that is designed to further reduce C5 accumulation by enhancing the cellular uptake and processing of C5–antibody immune complexes [31], thus enabling SC administration, with the possibility to be self-administered outside a healthcare setting. Additionally, due to its unique binding site on C5, crovalimab could still be efficacious in patients with the R885H C5 SNP [32,33].

Given the inherent limitations on the volume that can be effectively delivered via SC injection (typically not exceeding approximately 2 mL per injection [34]), achieving a liquid formulation that allows for a high concentration of antibody drugs is crucial. However, the challenge of high viscosity in such formulations poses a significant hurdle, making the development of practical and efficacious liquid formulations a complex endeavor [35]. Crovalimab’s design addresses this by prioritizing low viscosity, thereby enabling the delivery of therapeutic levels of the antibody in a limited volume, a critical factor for the success of SC antibody therapies.

Crovalimab is being evaluated in a clinical trial program that has included over 400 patients with PNH from over 30 countries [36,37,38,39,40]. Long-term follow-up results from the open-label adaptive four-part phase I/II COMPOSER trial (NCT03157635) showed that crovalimab was well tolerated, achieved sustained C5 inhibition, and maintained long-term efficacy over a 3-year median treatment duration in both C5 inhibitor-naive and C5 inhibitor-experienced patients with PNH [37]. The global, randomized, phase III COMMODORE 2 trial (NCT04434092) demonstrated that crovalimab was non-inferior to eculizumab for the co-primary efficacy endpoints of hemolysis control and transfusion avoidance, and the key secondary efficacy endpoints of breakthrough hemolysis and hemoglobin stabilization, in C5 inhibitor-naive patients [40]. Crovalimab was also shown to have a safety profile consistent with that of other C5 inhibitors [40], and patients experienced rapid, sustained, and clinically meaningful improvements in their fatigue and sustained improvements in functioning and PNH symptoms [40,41]. In the global, randomized, phase III COMMODORE 1 study (NCT04432584), crovalimab treatment maintained disease control, was well tolerated, and maintained the baseline levels of fatigue, functioning, and PNH symptoms in patients who switched from eculizumab [38,41]. Additionally, the majority of the patients in COMMODORE 1 and 2 who switched from eculizumab to crovalimab preferred crovalimab [38,40]. In the single-arm COMMODORE 3 (NCT04654468) trial conducted in China, crovalimab met its co-primary efficacy endpoints of hemolysis control and transfusion avoidance and was shown to be well tolerated [39]. Crovalimab is approved for the treatment of PNH [42,43,44,45]. As with any newly approved therapy, the ongoing monitoring of the patients in the COMMODORE studies and the collection of real-world evidence will inform the long-term safety profile of crovalimab and its optimal use in diverse patient populations.

This study provides a critical examination of crovalimab’s unique molecular features, highlighting its approach to enhancing the safety and efficacy in PNH treatment through the elimination of FcγR and C1q binding, alongside its innovative engineering to facilitate SC delivery. The findings not only contribute to the scientific understanding of antibody engineering but also pave the way for future advancements in patient-centric care, emphasizing the significance of this work in the broader context of therapeutic development.

## 2. Results

### 2.1. Creation Journey of Crovalimab and Key Antibody Engineering

**Figure 1:**
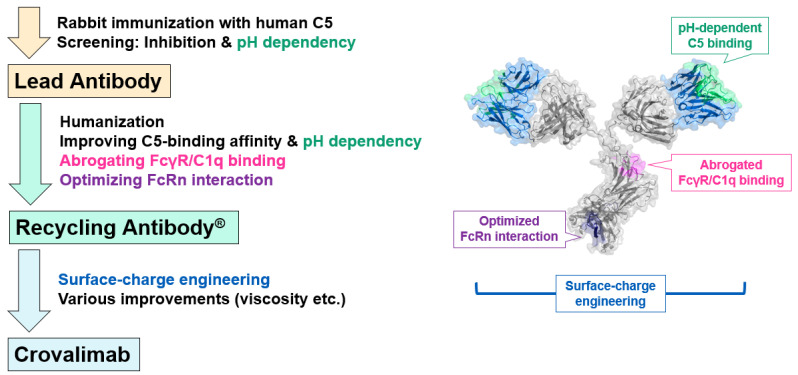
Schematic overview of the crovalimab creation journey. This diagram illustrates the key steps in the creation of this therapeutic agent, starting with the identification of the lead antibody, followed by the engineering of a recycling antibody and the subsequent enhancements that led to the final antibody, crovalimab. Sites where key antibody engineering technologies were applied are highlighted in the accompanying illustration of crovalimab. FcRn, neonatal fragment crystallizable receptor; FcγR, IgG-Fc receptor.

To create crovalimab, rabbit anti-C5 antibodies were produced by immunizing rabbits with human C5 (Figure 1) [31,33]. The screening process identified antibodies exhibiting neutralizing activity and pH-dependent C5 binding. The rabbit antibody CFA0305 was identified as the lead antibody due to its neutralizing activity and potent pH-dependent C5 binding properties. CFA0305 was then humanized and optimized for improved C5 binding activity, with high affinity at a neutral pH and fast dissociation at an acidic pH. Through the comprehensive substitution for multidimensional optimization (COSMO) process, the effects of single amino acid substitutions on the expression level, C5 binding properties, stability, etc., were evaluated. The antibody’s characteristics were then greatly improved by combining effective substitutions and evaluating a large number of variants. A human immunoglobulin (Ig)G1 constant region was engineered to abrogate binding to FcγRs and C1q to minimize potential safety risks and also enhance the affinity to FcRn at an acidic pH for a prolonged antibody plasma half-life. The recycling antibody created by existing technologies as described above partially suppressed the plasma C5 accumulation compared to the level observed with a conventional antibody but was not able to achieve the targeted SC delivery with long dosing intervals. This was because the recycling antibody could enhance C5 clearance by releasing C5 molecules in the endosome but was not able to improve the cellular uptake of immune complexes, resulting in significant C5 accumulation. Therefore, surface-charge engineering was performed to enhance the cellular uptake of immune complexes. Additionally, the developability and manufacturability (stability, solubility, viscosity, etc.) of the candidate antibodies was carefully monitored and improved. The final humanized and optimized antibody was later named crovalimab.

### 2.2. Engineered IgG1 Constant Region of Crovalimab

**Figure 2:**
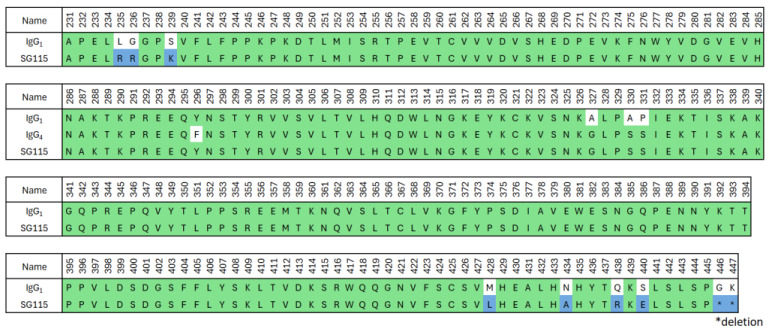
Amino acid alignment of crovalimab with human germline sequences. The amino acid sequence of the crovalimab Fc region (SG115) is shown together with the human IgG1 and IgG4 sequences. The green and blue regions in the sequence of SG115 indicate, respectively, the human-derived sequences and the artificial mutations introduced to improve the properties of the antibody. Ig, immunoglobulin.

The constant region of crovalimab’s heavy chain (SG115) was modified from human IgG1. Figure 2 shows the amino acid sequence of crovalimab’s Fc together with both the human IgG1 and IgG4 sequences, where green denotes the human-derived sequences and blue denotes the optimizing amino acids introduced in the sequence of SG115. Positions 235, 236, and 239 (Eu index) were engineered to abrogate binding to human FcγRs (hFcγRs) and human complement component C1q as a means of minimizing the potential safety risk of activating effector functions [31]. The sequence around positions 327–331 was derived from human IgG4, resulting in the silencing of C1q binding. Positions 428 and 434 were substituted to enhance FcRn binding for a prolonged antibody half-life. It has been reported that utilizing Fc engineering to increase FcRn binding causes enhanced binding to rheumatoid factors [46], which may potentially affect an antibody’s pharmacokinetics and immunogenicity. Substituting positions 438 and 440 effectively suppressed the enhanced rheumatoid factor binding. To reduce the C-terminal heterogeneity of the antibody’s heavy chain, the two C-terminal amino acids, glycine and lysine, were genetically removed.

### 2.3. FcγR and C1q Binding Properties of Crovalimab

**Figure 3:**
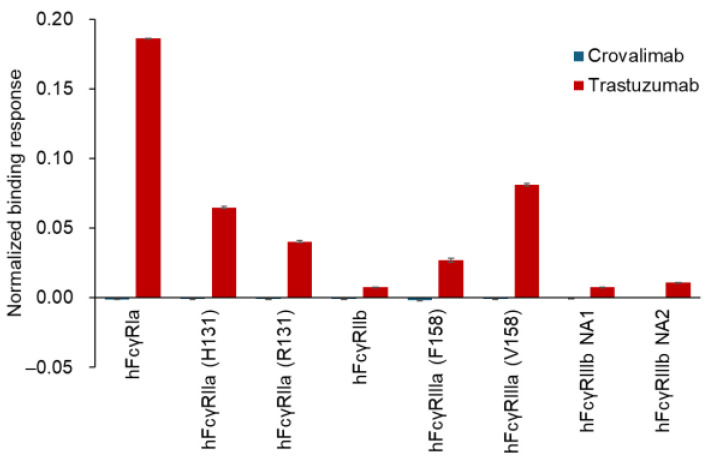
FcγR binding characteristics of crovalimab. SPR analysis of binding between hFcγRs and antibodies using the Biacore T200 system. Crovalimab and trastuzumab (control) were immobilized on a Protein A-coated sensor chip, and various hFcγRs, including polymorphic variants, were injected over them. Binding responses were normalized to 1 RU of antibody. Data represent mean ± SD (*n* = 3). Unlike trastuzumab, crovalimab showed no detectable binding to any hFcγRs, suggesting a minimal risk of activating effector functions through FcγRs. F, phenylalanine; H, histidine; hFcγR, human Fcγ receptor; NA, neutrophil antigen; R, arginine; RU, resonance units; SD, standard deviation; SPR, surface plasmon resonance; V, valine.

**Figure 4:**
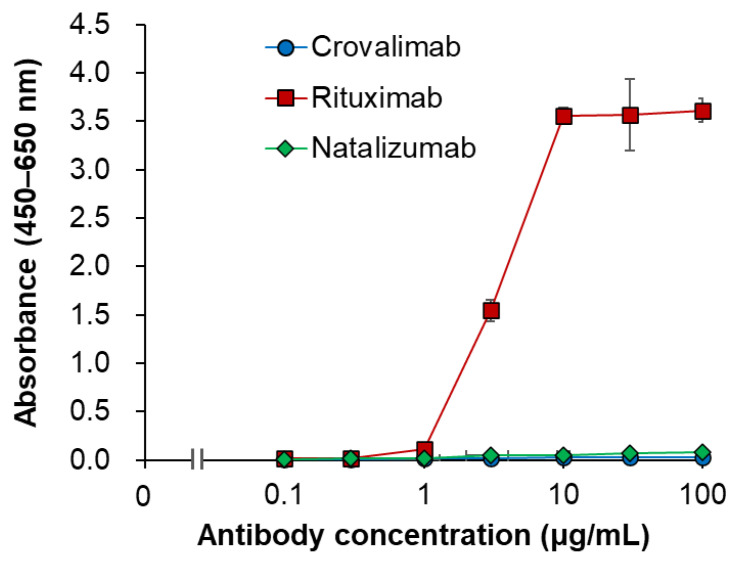
C1q binding characteristics of crovalimab. The binding activity of crovalimab, rituximab (positive control), and natalizumab (negative control) to human C1q protein was evaluated by ELISA. Antibodies were immobilized on 96-well plates, incubated with human C1q protein, and detected using anti-human C1q antibody. Data represent mean ± SD (*n* = 4). Points represent the average of the measured values for crovalimab (blue), rituximab (red), and natalizumab (green). Crovalimab and natalizumab showed comparably little binding to human C1q, in contrast to rituximab, indicating a minimal risk of complement activation through C1q binding with crovalimab despite its human IgG1 backbone. ELISA, enzyme-linked immunosorbent assay; SD, standard deviation.

The binding of crovalimab to hFcγRs was evaluated using surface plasmon resonance (SPR) analysis in comparison with trastuzumab, a humanized anti-HER2 monoclonal antibody (human IgG1), whose Fc region is known to bind to hFcγRs [47]. Although binding responses of trastuzumab to hFcγRs were observed, none were detected for crovalimab (Figure 3). The binding of crovalimab to the human complement C1q protein was also evaluated in comparison with the humanized monoclonal antibodies rituximab (anti-CD20, human IgG1, used as a positive control) and natalizumab (anti-α4-integrin, human IgG4, used as a negative control). In contrast to the binding activity of rituximab, crovalimab and natalizumab had comparably little binding to human C1q (Figure 4). Even though the backbone of crovalimab is human IgG1, crovalimab shows undetectable levels of binding to FcγRs and C1q, indicating that crovalimab has a minimal risk of activating effector functions through FcγRs or C1q.

### 2.4. C5 Binding Characteristics of Crovalimab

SPR analysis was carried out to evaluate and compare the C5 binding properties of approved anti-C5 antibodies under the same experimental conditions, to explore the unique C5 binding properties of crovalimab. This measurement showed that the dissociation constant (KD) for crovalimab binding to human C5 was 1.66 × 10^−10^ M at pH 7.4 and 1.32 × 10^−7^ M at pH 5.8 (Table 1, Figure S2A). The KDs at pH 7.4 and pH 5.8 were 1.38 × 10^−9^ M and 1.28 × 10^−7^ M, respectively, for a sequence-identical analog of ravulizumab (ravulizumab-SIA), and 1.42 × 10^−10^ M and 2.64 × 10^−9^ M, respectively, for a sequence-identical analog of eculizumab (eculizumab-SIA). To further explore the pH-dependent dissociation characteristics of the anti-C5 antibodies from human C5, a modified SPR assay was conducted, which incorporated an additional dissociation phase at pH 5.8 immediately following the standard dissociation phase at pH 7.4 (Figure S2B). These SPR analyses revealed that crovalimab binds strongly to C5 at the neutral pH of blood (similar to eculizumab-SIA) and rapidly dissociates at the acidic pH of endosomes (comparable to ravulizumab-SIA). The KD ratio between pH 5.8 and 7.4 for crovalimab is 796-fold, significantly higher than that of eculizumab-SIA (18.6-fold) and ravulizumab-SIA (92.4-fold), demonstrating its improved pH-dependent C5 binding property.

Crovalimab’s ability to neutralize C5 activity was not impacted by the R885H SNP, distinguishing it from eculizumab [33]. The epitope bound by eculizumab is affected by alterations at position 885. Similar to eculizumab, ravulizumab-SIA was unable to inhibit the activity of C5 variants carrying the R885H SNP, as demonstrated in the liposome lysis assay (Figure S3A). To further illustrate crovalimab’s interaction with C5, especially in the context of the R885H SNP, a structural model of the crovalimab–C5–eculizumab complex is shown in Figure S3B. This model provides supplementary evidence that crovalimab and eculizumab/ravulizumab bind to different epitopes on C5, with crovalimab’s binding site not overlapping with the position affected by the R885H SNP.

### 2.5. Effect of pH Dependency and Surface-Charge Engineering on C5 Clearance

**Figure 5:**
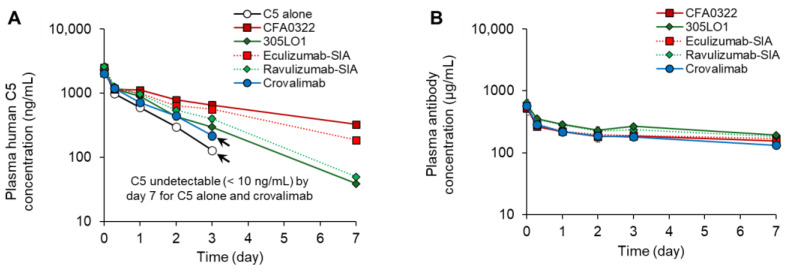
Time course profiles of (**A**) human C5 concentration and (**B**) anti-C5 antibody concentration in human FcRn transgenic mice after IV administration of human C5 (0.1 mg/kg) with or without an anti-C5 antibody (20 mg/kg). Data are presented as mean ± SD with *n* = 3. The limit of quantification was 10 ng/mL for C5 and 0.02 µg/mL for anti-C5 antibodies, and values below this threshold are not displayed. Notably, the plasma human C5 concentrations fell below the detection limit by day 7 in both the C5 alone and crovalimab-treated groups, leading to the truncation of the data lines at day 3, as indicated by the arrows. This observation highlights the effectiveness of recycling antibody technology and surface-charge engineering in accelerating C5 clearance. The plasma concentrations of all antibodies remained similar throughout the study period. Reprinted from Sampei Z, et al. [31] and Fukuzawa T, et al. [33]. Creative Commons Attribution 4.0 International License (https://creativecommons.org/licenses/by/4.0/ accessed on 1 October 2024). FcRn, neonatal Fc receptor; IV, intravenous; SD, standard deviation; SIA, sequence-identical analog.

To enhance the clearance of C5–antibody immune complexes and suppress C5 accumulation, crovalimab was engineered to modify the surface charge without compromising the extended plasma half-life [33]. The experimental isoelectric point of crovalimab (produced from HEK293 cells) was found to be 9.17. Cation exchange chromatography (CIEX) analysis showed the formation of crovalimab–C5 immune complexes (Figure S4). We previously analyzed the relationship between the maximum level of C5 accumulation in cynomolgus monkeys and the CIEX retention time (an indicator of the charge characteristics) of immune complex-1 (IC-1), consisting of one antibody and one C5 molecule, using 305LO1-SG407 (a pH-dependent CFA0305 variant with FcRn binding enhancement but without charge engineering) and its derivatives with an engineered surface charge [33]. Following our earlier work, we analyzed crovalimab and found a consistent correlation between the CIEX retention time of IC-1 and the maximum C5 accumulation in cynomolgus monkeys. The effect of the pH dependency and surface-charge engineering on C5 clearance and the pharmacokinetics of several anti-C5 antibodies, including crovalimab, was demonstrated in a human FcRn transgenic mouse model [31,33]. The anti-C5 antibodies evaluated were eculizumab-SIA, CFA0322 (a high-affinity antibody without pH dependency, obtained through rabbit immunization), 305LO1 (a pH-dependent recycling antibody without FcRn binding enhancement), ravulizumab-SIA (engineered with pH dependency and FcRn binding enhancement, but without charge modification), and crovalimab. The results showed that 305LO1 and ravulizumab-SIA exhibited faster C5 clearance than eculizumab-SIA and CFA0322, and the surface-charge-engineered crovalimab demonstrated even faster C5 clearance, reaching below the detection limit (10 ng/mL) at day 7 (Figure 5A), while the plasma concentrations of all antibodies were similar (Figure 5B), reconfirming the effectiveness of recycling antibody technology and charge engineering in accelerating C5 clearance.

### 2.6. Efficiency of C5 Inhibition by Crovalimab

Regarding the analysis of the approved dosing regimen for maintenance therapy in PNH using crovalimab, eculizumab, and ravulizumab, Table 2 quantifies the average amount of C5 neutralized by a single molecule of each antibody. This assessment is crucial in understanding the mechanism of crovalimab compared with its counterparts. The analysis calculated the ratio of the daily endogenous production rate of C5 to the daily equivalent dosage of each antibody (C5 production per day/antibody dose per day), factoring in the SC bioavailability for crovalimab. For eculizumab, with a maintenance dose of 900 mg Q2W, the ratio is 2.07, indicating that, on average, each antibody inhibits approximately two molecules of C5. In the case of ravulizumab, administered at 3300 mg Q8W, the ratio increases slightly to 2.25, suggesting that each antibody neutralizes just over two molecules of C5. Conversely, crovalimab, given at a maintenance dose of 680 mg every 4 weeks (Q4W), shows that each antibody molecule inhibits 7.42 molecules of C5, highlighting its enhanced efficiency in neutralizing C5.

### 2.7. Viscosity Potential of Crovalimab

**Figure 6:**
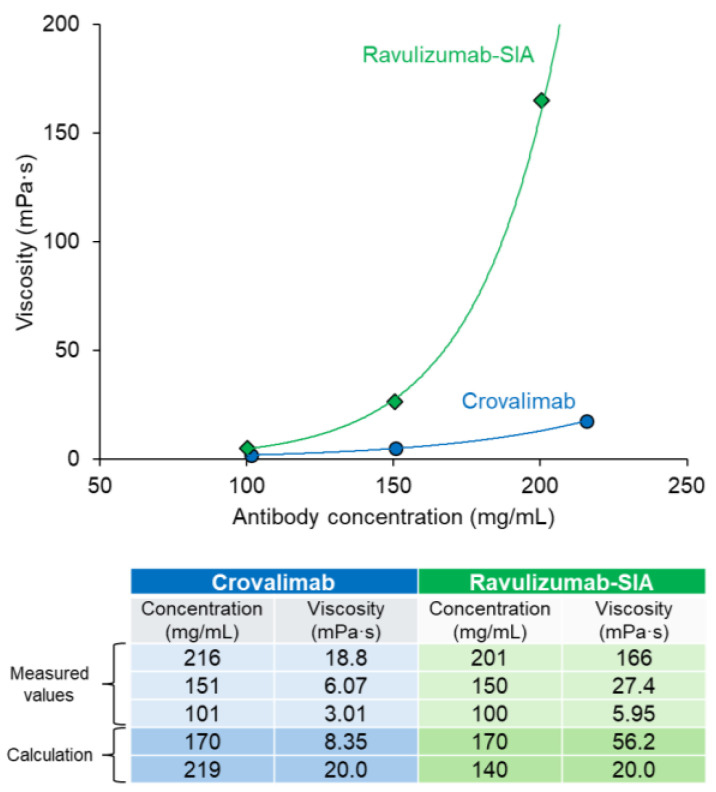
Relationship between antibody concentration and viscosity within the same formulation: a 20 mM histidine, 150 mM arginine/aspartic acid buffer (pH 6.0). The measured values of the antibody concentration and viscosity are plotted in the graph and presented in the table, which also includes the calculated viscosity at 170 mg/mL and the antibody concentration at which the viscosity reaches 20 mPa·s. Crovalimab demonstrated lower viscosity compared with ravulizumab-SIA at equivalent concentrations, enabling a high-concentration formulation (170 mg/mL) suitable for SC injection. Viscosity measurements were conducted at 25 °C using an EMS-01S viscometer. Each data point represents the mean of three measurements (*n* = 3), with each measurement being the average of 10 consecutive readings. The buffer viscosity was subtracted from each measurement. An exponential approximation curve was applied to the corrected values for precise modeling, with an R^2^ value approaching 1. SIA, sequence-identical analog.

As the viscosity must be low to achieve an SC-injectable antibody with a high concentration in a liquid formulation, the antibody’s structure was carefully engineered, considering the charge distribution, etc., to lower the viscosity [35]. Previously reported data indicate an upper viscosity limit of approximately 50 mPa·s for the SC administration of 1 mL of an antibody suspension via a 26-gauge syringe over 20 s [49]. The viscosity of the antibody candidates was periodically measured, and the viscosity was carefully considered when selecting the candidate antibody for development. The final antibody chosen, crovalimab, showed an acceptable viscosity level, which is necessary to enable the high-concentration antibody formulation required for SC injection. When both antibody concentration and formulation were the same, crovalimab showed lower viscosity than ravulizumab-SIA (Figure 6). At a concentration of approximately 150 mg/mL, the viscosity of crovalimab was 6.07 mPa·s vs. 27.4 mPa·s for ravulizumab-SIA. At this acceptable and injectable level of viscosity, a high-antibody-concentration formulation of 170 mg/mL was successfully achieved for crovalimab.

## 3. Discussion

This study elucidates the innovative molecular design and function of the recently approved crovalimab, carefully engineered to enable its optimized Q4W maintenance SC dosing regimen, thereby addressing critical unmet needs in the treatment of PNH [50], where lifelong management is required. Crovalimab was created through a process including rabbit immunization, humanization, recycling antibody generation by improving pH-dependent antigen binding and optimizing FcRn interaction, and surface-charge engineering (Figure 1).

Despite its constant region being derived from human IgG1 (Figure 2), which is known to engage hFcγR and human C1q, crovalimab was engineered to eliminate these interactions, thus minimizing the potential safety concerns (Figure 3 and Figure 4). This is in contrast to human IgG2 and IgG4, both of which possess hFcγR binding capabilities [51].

Central to crovalimab’s functionality is its engineered pH-dependent C5 binding, as presented in Table 1. This feature enables high-affinity binding in the neutral pH environment (comparable to eculizumab-SIA) and significantly weaker binding under acidic conditions (comparable to ravulizumab-SIA). Such a characteristic ensures that crovalimab can effectively neutralize C5 in the blood circulation while facilitating rapid release from C5 in the acidic endosomes. Moreover, crovalimab incorporates innovative surface-charge engineering to enhance the cellular uptake of the C5–crovalimab immune complexes, thereby promoting more efficient C5 clearance from the blood circulation compared with existing therapies such as eculizumab and ravulizumab. This charge engineering is crucial because the recycling antibody technology alone was not sufficient to achieve our targeted SC delivery with long dosing intervals.

**Figure 7:**
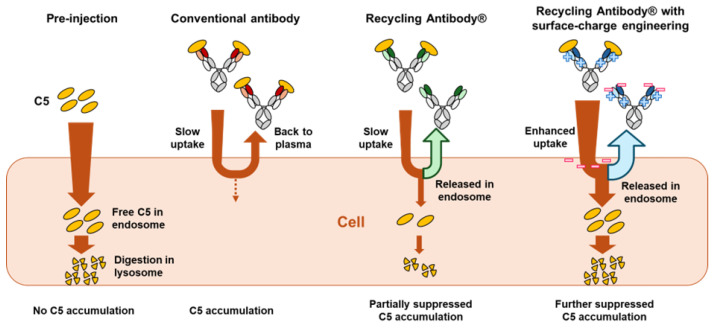
Cellular uptake of immune complexes and subsequent impact on C5 accumulation across various antibody types, including conventional non-pH-dependent antibodies, recycling antibodies without surface-charge engineering, and recycling antibodies with surface-charge engineering (such as crovalimab).

Figure 7 provides a comparative visualization of the cellular uptake of the immune complex and the impact on C5 accumulation among various antibody types, including conventional non-pH-dependent antibodies, recycling antibodies without charge engineering, and recycling antibodies that have surface-charge engineering (i.e., crovalimab). Non-pH-dependent conventional antibodies can cause C5 accumulation in the plasma, due to the formation of immune complexes between C5 and the antibody, which decreases C5 clearance [31]. The introduction of a pH-dependent C5 binding property can partially suppress this antigen accumulation. Additionally, the novel engineering of the surface charge, as employed in crovalimab, enhances the cellular uptake of immune complexes, further reducing C5 accumulation without compromising the prolonged antibody pharmacokinetics. This advanced mechanism promotes the expedited clearance of C5, thereby reducing the dosage requirements.

In previous studies, the impact of antibody administration on C5 clearance, and consequently on C5 accumulation, was evaluated through experiments involving the simultaneous administration of human C5 and antibodies in human FcRn transgenic mice. These studies demonstrated that crovalimab exhibited faster C5 clearance compared with a non-pH-dependent antibody (CFA0322) [33] and that a pH-dependent antibody (305LO1) showed faster C5 clearance than eculizumab [31]. However, these studies included no direct comparisons between 305LO1 and crovalimab or between ravulizumab and crovalimab. The results from the mouse experiments presented in this paper revealed that pH-dependent antibodies, such as 305LO1 and ravulizumab-SIA, demonstrated faster C5 clearance compared with conventional antibodies like CFA0322 and eculizumab-SIA, but crovalimab facilitated even more rapid C5 clearance (Figure 5). Although the effect of charge engineering on C5 clearance had been previously shown in cynomolgus monkey experiments [31], direct comparisons between crovalimab, eculizumab, and ravulizumab could not be conducted in cynomolgus monkeys due to the low cross-reactivity of eculizumab and ravulizumab with the cynomolgus monkey C5 [52]. Hence, in this study, we confirmed the effectiveness of charge engineering by comparing crovalimab with existing antibodies in a mouse model using human C5, highlighting the advantages of crovalimab.

In addition to the preclinical findings presented, it is pivotal to consider the impact of these molecular designs in a clinical setting. Results from Study 301 have indicated that eculizumab and ravulizumab accumulate C5 to comparable levels in patients (183.3 [standard deviation (SD) 36.5] µg/mL and 196.1 [SD 38.5] μg/mL, respectively, at day 183) [53]. This suggests that, despite their effectiveness in treating PNH, there remains room for improvement in minimizing C5 accumulation, which is crucial for the comprehensive management of the disease. Furthermore, data from the COMPOSER trial, where patients transitioned from eculizumab to crovalimab, show a subsequent decrease in the C5 concentration. This substantial reduction underscores the practical effectiveness of crovalimab’s molecular design in mitigating C5 accumulation more effectively in a clinical context. Crovalimab also demonstrates an extended plasma half-life of approximately 2 months in patients, which is comparable to that of ravulizumab [53]. These clinical observations substantiate the molecular innovations behind crovalimab, specifically its charge engineering, pH-dependent C5 binding, and optimized FcRn interaction, as not only theoretically advantageous but also beneficial in a clinical setting. With its lower C5 accumulation and prolonged plasma half-life, crovalimab achieves highly efficient C5 neutralization, enabling lower dosage requirements.

To quantify the efficiency with which each antibody molecule neutralizes C5 molecules, we analyzed the ratio of the endogenous production rate of C5 to the administered dosage of each antibody (Table 2). This analysis revealed marked differences in the efficiency of C5 neutralization by eculizumab, ravulizumab, and crovalimab, with average values of 2.07, 2.25, and 7.42, respectively. These findings highlight the unique molecular design of crovalimab, which contributes to its distinctive neutralization capacity. It is crucial to note, however, that while our analysis provides valuable insights into the efficiency of these antibodies in neutralizing C5, there are inherent limitations to this comparison. The dosing regimens of these antibodies vary; for example, ravulizumab is administered at a relatively high dose of 3300 mg Q8W, which could lead to a prolonged period of antibody concentrations exceeding those of C5. This could result in a proportion of antibodies being eliminated without neutralizing C5, thereby affecting the average number of C5 molecules neutralized per antibody. Such factors must be considered when interpreting the C5 neutralization capabilities of each antibody included in this analysis.

Overall, our findings indicate that crovalimab’s design allows for a substantial reduction in the dosage needed for efficient C5 inhibition, reducing the administration frequency compared with eculizumab and enabling SC maintenance dosing at 680 mg Q4W. This reduction in crovalimab’s dosage can be attributed to crovalimab’s enhanced ability to remove C5, thus preventing its accumulation and ensuring sustained neutralization over time.

In population screening studies, C5 R885H SNPs were identified in approximately 3.2–3.8% of healthy individuals of Japanese descent [29,54] and have also been reported in people of European descent and no Asian ancestry [55]. Previous clinical data have indicated that patients with the C5 R885H SNP encounter challenges in achieving optimal therapeutic outcomes with eculizumab and ravulizumab [22,29]. In contrast, crovalimab was able to neutralize the R885H SNP variant of C5 in vitro [33], unlike eculizumab and ravulizumab. Moreover, an analysis of four patients of Asian descent who had the C5 R885H SNP in the phase I/II COMPOSER study [32,36] indicated that there was a rapid reduction in LDH and that the LDH level was sustained.

In the exploration of crovalimab’s molecular design, one of the focal points has been its optimized low viscosity, which not only facilitates manufacturing but also allows for the formulation of a high-concentration antibody solution suitable for SC delivery in reduced volumes. Furthermore, the low viscosity of SC drugs is essential in optimizing the injectability by reducing the force and time required for administration. However, achieving low-viscosity antibody formulations at high concentrations is particularly difficult because the viscosity increases with the antibody concentration, and it is challenging to lower the viscosity potential by antibody engineering without compromising their pharmacologic properties. This study compared the viscosity potential of crovalimab and ravulizumab-SIA, using an identical liquid formulation for analysis (Figure 6). Our findings indicate that crovalimab exhibits an acceptable level of viscosity, enabling a drug product concentration of 170 mg/mL for SC injection. At this concentration, administering a total volume of 4 mL across two injections delivers 680 mg of crovalimab, which is adequately high to ensure effective C5 neutralization. The successful reduction in crovalimab’s viscosity aligns with the initial design objectives and represents a significant advancement in the development of SC-injectable antibody therapies. Crovalimab’s success in combining a high concentration with manageable viscosity showcases the pivotal role of molecular design in addressing SC delivery challenges, potentially leading to treatments that are both easier to administer and more patient-friendly.

Although this study provides valuable insights into the molecular design and function of crovalimab, several limitations should be acknowledged. Our findings are primarily based on preclinical studies in mice and cynomolgus monkeys, whose physiological responses may differ from those of humans. As discussed earlier, the comparison of the C5 inhibition efficiency is strongly influenced by the dosing regimens, likely underestimating the full neutralization capacity of crovalimab due to its administration, aimed at maintaining sufficient levels in patients. The viscosity evaluations were conducted using identical formulations for a fair comparison, but the optimal formulations may vary between molecules, possibly altering the results. Despite these limitations, it is noteworthy that the clinical efficacy and safety of crovalimab have been demonstrated in patients, leading to its approval as an SC formulation. This clinical success indicates that the molecular design innovations of crovalimab have been effectively translated to real-world therapeutic applications. Future studies should focus on the long-term clinical outcomes and effects on rare C5 variants to further elucidate the full therapeutic potential of crovalimab in diverse patient populations.

## 4. Materials and Methods

### 4.1. Preparation of C5, FcγRs, and Antibodies

Recombinant human C5 and cynomolgus monkey C5, as well as the extracellular regions of hFcγRs, were expressed in FreeStyle 293-F cells (Thermo Fisher Scientific Inc., Waltham, MA, USA) and purified as previously described [33,56]. Crovalimab, trastuzumab, and rituximab were sourced from Chugai Pharmaceutical Co., Ltd./F. Hoffmann-La Roche Ltd. (Basel, Switzerland), while natalizumab was obtained from Biogen Inc. (Cambridge, MA, USA). Eculizumab-SIA and ravulizumab-SIA were produced in-house by purification from the culture supernatants of transfected cells following standard protocols, which included affinity chromatography and the necessary subsequent purification steps, such as gel filtration, to achieve the desired purity levels.

### 4.2. FcγR Binding Assay

Crovalimab and trastuzumab were each prepared at a concentration of 5 μg/mL in a buffer comprising 50 mM sodium phosphate, 150 mM NaCl, and 0.005% Surfactant P20, pH 7.4. These antibodies were immobilized on a sensor chip precoated with recombinant Protein A using the Biacore T200 system (Cytiva, Marlborough, MA, USA), at a flow rate of 10 μL/min, for 2 min. Subsequently, various hFcγRs (hFcγRIa, hFcγRIIa [H131], hFcγRIIa [R131], hFcγRIIb, hFcγRIIIa [F158], hFcγRIIIa [V158], hFcγRIIIb NA1, and hFcγRIIIb NA2), prepared in-house, were injected over the immobilized antibodies to assess the binding interaction. The binding assessment was performed in triplicate under consistent flow conditions. To establish negative controls, the FcγRs and buffer were directly injected into control flow cells without prior antibody capture. The extent of the binding interaction was quantified using the Biacore T200 Evaluation Software (Cytiva), with the data normalized to 1 resonance unit (RU) of captured antibody.

### 4.3. C1q Binding Assay

Antibody solutions were prepared by diluting crovalimab, rituximab, and natalizumab in phosphate-buffered saline. These solutions were then immobilized onto 96-well plates and incubated at 4 °C overnight. After immobilization, the plates were washed and subjected to blocking for 2 h at room temperature. The assay continued with the addition of human C1q protein (Merck Millipore Corporation, Burlington, MA, USA) diluted to 3 μg/mL, followed by 1 h incubation. Detection was performed using a sheep anti-human C1q antibody (Bio-Rad Laboratories, Inc., Hercules, CA, USA), with the subsequent addition of 3,3′,5,5′-tetramethylbenzidine chromogen solution (Thermo Fisher Scientific Inc.) for 10 min. The reaction was stopped, and the absorbance was measured at 450 nm, using 650 nm as a reference wavelength. The absorbance data were analyzed to quantify C1q binding.

### 4.4. C5 Binding Kinetics Analysis by Surface Plasmon Resonance

The kinetic binding properties of anti-C5 antibodies to recombinant human C5 under physiological (pH 7.4) and acidic (pH 5.8) conditions were evaluated at 37 °C using the Biacore T200 system (Cytiva). For this purpose, a goat anti-human IgG (Fc) polyclonal antibody (KPL #01-10-20) was immobilized onto a CM4 sensor chip utilizing an amine coupling kit (Cytiva), following the manufacturer’s recommended protocol. Both the antibodies and analytes were prepared in N-(2-acetamido)-2-aminoethanesulfonic acid (ACES) buffer (20 mM ACES, 150 mM NaCl, 1.2 mM CaCl_2_, 0.05% Tween 20, 0.005% NaN_3_) adjusted to either pH 7.4 or pH 5.8. Antibodies were captured on the sensor surface using the anti-Fc approach, with captures levels typically ranging between 50 and 80 RU. Recombinant human C5 was serially diluted in a three-fold manner, starting at 27 nM for assays at pH 7.4 and 135 nM for assays at pH 5.8. The sensor surface was regenerated with 20 mM HCl and 0.01% Tween 20. Data analysis was performed using the Biacore T200 Evaluation Software (Cytiva), applying a 1:1 binding model for fitting. To further explore the pH-dependent dissociation characteristics of the anti-C5 antibodies from human C5, a modified SPR assay was conducted. This modification entailed incorporating an additional dissociation phase at pH 5.8, immediately following the standard dissociation phase at pH 7.4. This approach aimed to assess the pH-dependent binding stability of the antibody–antigen complexes initially formed at pH 7.4.

### 4.5. Neutralizing Activity of Anti-C5 Antibodies

The inhibition of C5 variants by ravulizumab-SIA (prepared in-house) was assessed by measuring the membrane attack complex activity using a liposome lysis assay, as previously described [33]. Briefly, 20 µL of diluted ravulizumab-SIA, 20 µL of cell culture medium containing recombinant human C5 variants (V145I, R449G, V802I, R885H, R928Q, D966Y, S1310N, and E1437D at 0.02–0.03 µg/mL), and 10 µL of C5-deficient human serum (C1163, Sigma-Aldrich Co. LLC, St. Louis, MO, USA) were mixed and incubated at 37 °C for 30 min in a plate. Sensitized liposomes and the substrate solution were then added to each well and the final mixture was incubated at 37 °C for 150 min. The optical density was measured at 340 nm. Data analysis was performed using the GraphPad Prism software (version 10.2, GraphPad Software Inc., Boston, MA, USA)

### 4.6. Structure Modeling

A structural model of the complexes comprising the crovalimab fragment antigen-binding region (Fab), eculizumab Fab, and human C5 was constructed based on the structural information from Protein Data Bank entries 5B71 (crovalimab–C5 MG1 domain) and 5I5K (eculizumab–C5). The complexes were superposed by aligning the MG1 domain of each complex, using the E chain of 5B71 and the B chain of 5I5K. Missing residues were modeled by a loop modeling method. These modeling processes were carried out using the Molecular Operating Environment (MOE) software package (version 2022.02) and the figure was generated with PyMOL (version 2.5.7, Schrödinger, Inc., New York, NY, USA).

### 4.7. Simulation of Effect of C5–Antibody Complex Clearance on C5 Accumulation

The plasma total antibody, total target antigen, and free target antigen concentrations after biweekly SC administration at 250 mg were simulated using a dynamic target-mediated drug disposition model described previously [57]. A one-compartment model with first-order absorption and elimination was used to simulate the plasma antibody concentration. The geometric mean of apparent CL (4.78 mL/day/kg) [58] and absorption rate constant (ka; 0.287/day) [57] after SC injection in humans were used for the simulation. For the target antigen parameters, the C5 parameters estimated from the plasma C5 concentration–time profile after IV injection in humans [48] were used as the model target antigen. The binding affinity was assumed to be 0.1 nM (kon: 100 [/day/nM], koff: 10 [/day]). All simulations were performed using the SAAM II software (version 1.2, Nanomath LLC, Spokane, WA, USA).

### 4.8. In Vivo Study Using Human FcRn Transgenic Mice

Animal care and experiments were performed in accordance with the guidelines for the care and use of laboratory animals at Chugai Pharmabody Research Pte. Ltd. (CPR, Singapore). The experimental protocols were approved by the Institutional Animal Care and Use Committee (IACUC) of CPR. All experimental procedures were carried out by licensed personnel. Solutions containing human C5 (0.1 mg/kg) only or a mixture of human C5 (0.1 mg/kg) and anti-human C5 antibody (20 mg/kg) were administered by IV injection into human FcRn homozygous transgenic mice, line #32 (B6.16 mouse FcRn−/−, human FcRn transgenic line 32+/+ mouse, Jackson Laboratories, Bar Harbor, ME, USA). Three mice (*n* = 3) were used for each experimental group. The concentration of total human C5 or anti-human C5 antibody in mouse plasma was measured by an electrochemiluminescence assay, as previously described [33].

### 4.9. CIEX Analysis

CIEX analysis was performed on a mixture of crovalimab and C5 molecules (antibody–antigen ratio of 1:1 [Fab–antigen ratio of 2:1]) using a ProPac WCX-10 high-performance liquid chromatography (HPLC) column (Thermo Fisher Scientific) with a particle size of 10 µm, diameter of 4 mm, and length of 250 mm, on an Alliance HPLC system (Waters Corporation, Milford, MA, USA). Mobile phase A was 25 mM 2-morpholinoethanesulphonic acid (MES) at pH 7.0 and mobile phase B was 25 mM MES and 250 mM sodium chloride at pH 7.0, at a flow rate of 0.5 mL/min. A linear gradient was run from 10% to 70% of mobile phase B for 50 min, and the column was then washed at 100% of mobile phase B for 5 min and further equilibrated at 10% of mobile phase B for 5 min. Elution was monitored by UV absorbance at 280 nm. Data analysis was performed using the Empower 3 software (Waters).

### 4.10. Viscosity Assessment

Viscosity measurements were conducted using an EMS-01S viscometer (Kyoto Electronics Manufacturing Co., Ltd., Kyoto, Japan) at 25 °C. Antibody solutions were prepared at approximately 200 mg/mL or higher in a 20 mM histidine, 150 mM arginine/aspartic acid buffer (pH 6.0), and then serially diluted for analysis. Each diluted sample was measured three times, with each measurement being the average of 10 consecutive readings, following the manufacturer’s calibration protocol. To refine our analysis, the viscosity of the buffer alone was subtracted from each measurement. These adjusted viscosity measurements were then plotted against their respective antibody concentrations. An exponential approximation curve was applied to these corrected values, achieving a highly precise model with an R^2^ value approaching 1. Based on the model, the viscosity corresponding to an antibody concentration of 170 mg/mL and the antibody concentration corresponding to a viscosity of 20 mPa·s were calculated.

### 4.11. Quantification of Efficiency of C5 Neutralization

The C5 production rate was calculated by dividing the plasma C5 clearance by the plasma C5 concentration. Plasma C5 clearance was estimated by fitting the reported plasma C5 concentration–time profile data after IV injection in humans using a two-compartment model [48]. The plasma C5 concentration was based on a previous report [36]. The quantity of antibodies (in nmol) required for maintenance dosing was determined by dividing the daily equivalent dosage (in ng), factoring in the SC bioavailability specifically for crovalimab, by the molecular weight of the antibody. This analysis calculated the ratio of endogenous C5 production per day to the daily equivalent dosage of each antibody, enabling the assessment of the efficiency of C5 neutralization by anti-C5 antibodies.

## 5. Conclusions

The findings of this study emphasize crovalimab’s innovative molecular design, which notably reduces C5 accumulation and enhances C5 neutralization, leading to lower dosage requirements for patients. Achieved through the integration of recycling antibody technology with novel surface-charge engineering, crovalimab is distinct from existing recycling antibodies. Additionally, its optimized low viscosity allows for the easier administration of high-concentration antibody formulations. This reduced dosage and low viscosity facilitate the SC administration of crovalimab. Consequently, as supported by recent approvals, crovalimab potentially presents a more convenient treatment option for patients with PNH, offering a four-weekly SC administration regimen and the possibility to be self-administered outside a supervised healthcare setting.

## Figures and Tables

**Table 1:** Binding kinetics of crovalimab, ravulizumab-SIA, and eculizumab-SIA against recombinant human C5 at pH 7.4 and pH 5.8, as measured by SPR at 37 °C. Experiments were performed using the Biacore T200 system with antibodies captured on a CM4 sensor chip. Data were analyzed using a 1:1 binding model. KD, dissociation constant; SIA, sequence-identical analog; SPR, surface plasmon resonance.

Antibody	pH 7.4			pH 5.8			Ratio of K_D_ at pH 5.8/pH 7.4
	ka (1/Ms)	kd (1/s)	K_D_ (M)	ka (1/Ms)	kd (1/s)	K_D_ (M)	
Crovalimab	7.41 × 10^5^	1.23 × 10^−4^	1.66 × 10^−10^	1.25 × 10^5^	1.64 × 10^−2^	1.32 × 10^−7^	796
Ravulizumab-SIA	6.07 × 10^5^	8.39 × 10^−4^	1.38 × 10^−9^	2.64 × 10^5^	3.38 × 10^−2^	1.28 × 10^−7^	92.4
Eculizumab-SIA	1.33 × 10^6^	1.89 × 10^−4^	1.42 × 10^−10^	1.45 × 10^6^	3.82 × 10^−3^	2.64 × 10^−9^	18.6

**Table 2:** Quantification of average number of C5 molecules processed by approved antibodies in PNH treatment, based on an analysis of approved dosing regimens. Calculations considered the endogenous C5 production rate, plasma C5 clearance, and antibody dosing, with SC bioavailability for crovalimab. PNH, paroxysmal nocturnal hemoglobinuria; Q2W, every 2 weeks; Q4W, every 4 weeks; Q8W, every 8 weeks; SC, subcutaneous. * Considering 73.7% SC bioavailability of crovalimab.

	Human C5		
C5 concentration [36]	134 μg/mL (704 nM)		
C5 clearance [48]	17.0 mL/day/kg		
C5 production per day	12.0 nmol/day/kg (897 nmol/day for 75 kg body weight)		
	Eculizumab	Ravulizumab	Crovalimab
Maintenance dosing regimen [22,23,38,40]	900 mg IV Q2W	3300 mg IV Q8W	680 mg SC (501 mg bioavailable *) Q4W
Antibody dose per day	64.3 mg/day (434 nmol/day)	58.9 mg/day (398 nmol/day)	17.9 mg/day (121 nmol/day)
Ratio of C5 production per day to antibody dose per day	897/434 = 2.07 2.07 C5 molecules per antibody	897/398 = 2.25 2.25 C5 molecules per antibody	897/121 = 7.42 7.42 C5 molecules per antibody

## Data Availability

All data generated or analyzed during this study are included in this published article.

## Supplementary Materials

The following supporting information can be downloaded at: https://www.mdpi.com/article/10.3390/ijms252111679/s1.
