# Supplementary material for: Beyond Recycling Antibodies: Crovalimab’s Molecular Design Enables Four-Weekly Subcutaneous Injections for PNH Treatment
Source: Int J Mol Sci. 2024 Oct 30;25(21):11679. doi: 10.3390/ijms252111679 (PMC11546984; doi:10.3390/ijms252111679)

Supplementary Materials

Figure S1. Simulation analysis to show the significance of the complex clearance and antigen accumulation. Kinetics of (**A**) antibody concentration over time, which is identical across all antibodies (black). Kinetics of (**B**) total antigen level and (**C**) free antigen level over time under conditions where the ratio of antigen clearance (before antibody injection) to complex clearance was set at 1 (blue), 2 (green), and 3 (red).

Ag, antigen; CL, clearance; IC, immune complex.

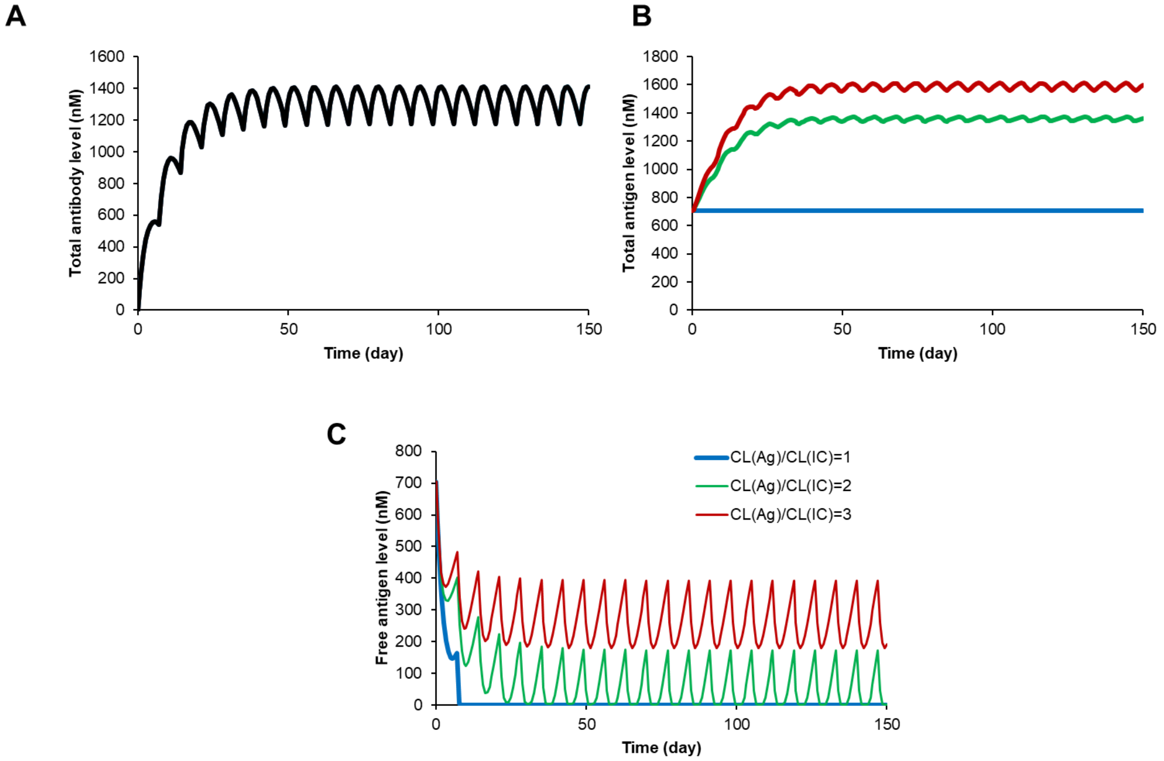

Figure S2. C5-binding characteristics. (**A**) SPR sensorgrams of human C5 binding to crovalimab, eculizumab-SIA, and ravulizumab-SIA at pH 7.4 and pH 5.8. (**B**) Modified SPR assay showing anti-C5 antibody binding kinetics to human C5, with association and dissociation at pH 7.4 followed by dissociation at pH 5.8.

RU, resonance units; SIA, sequence-identical analog; SPR, surface plasmon resonance.

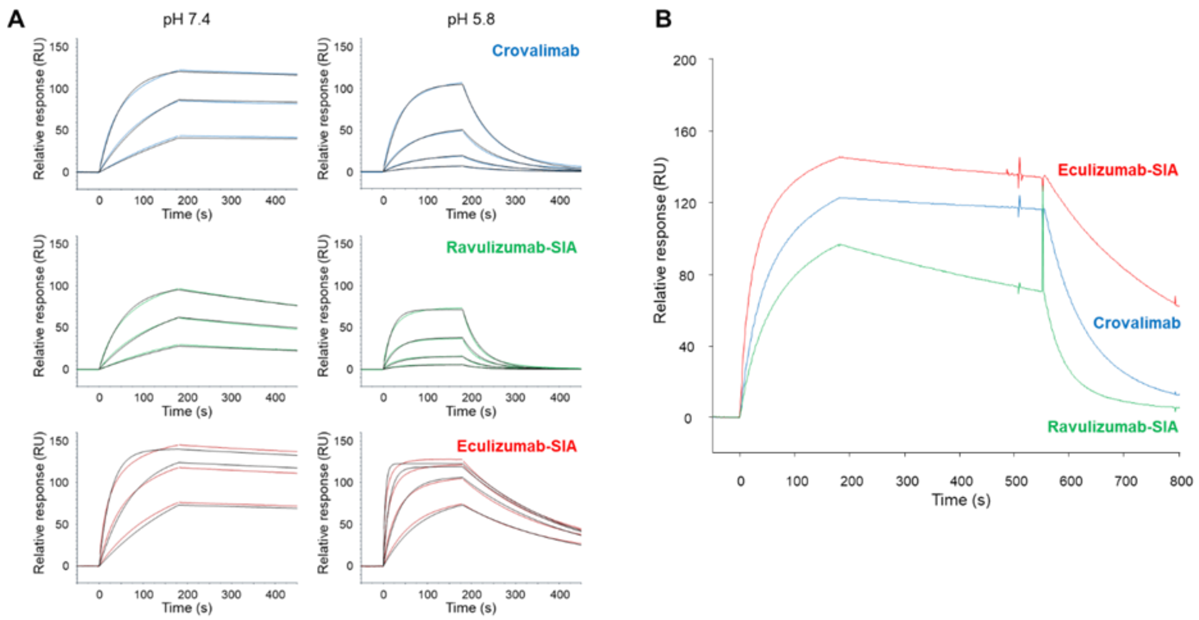

Figure S3. C5 inhibitory activity. (**A**) Neutralizing activity of ravulizumab-SIA on C5 variants with non-synonymous SNPs. Data are presented as mean ± SD. (**B**) Structural model of the crovalimab-C5-eculizumab complex, highlighting the distinct epitope binding sites of crovalimab compared with eculizumab/ravulizumab. Arginine at position 885 (R885) is shown in green stick representation and indicated with an arrow.

MAC, membrane attack complexes; SD, standard deviation; SIA, sequence-identical analog; SNP, single nucleotide polymorphism; WT, wild type.

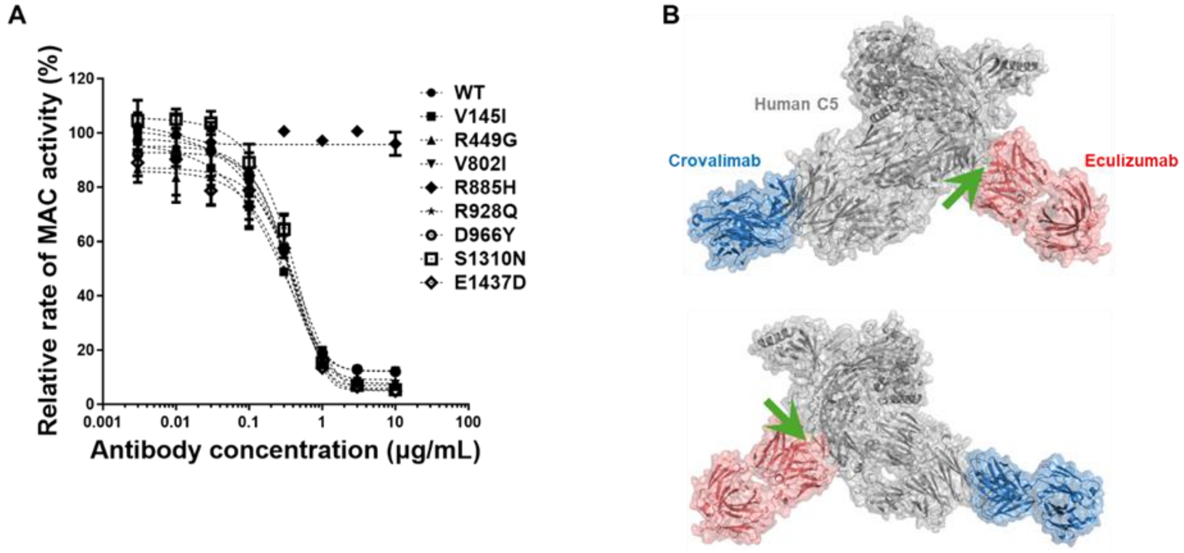

Figure S4. CIEX analysis of C5 (black line) and C5-crovalimab mixture (blue line).

Ab, antibody; AU, absorbance unit; CIEX, cation-exchange chromatography; IC, immune complex.

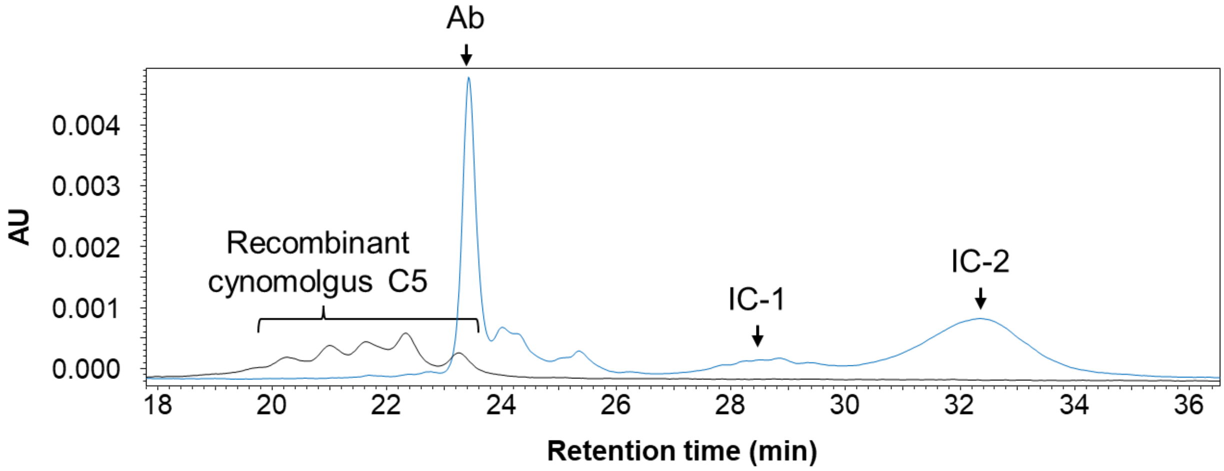

Supplement: Supplementary file 1 [file ijms-25-11679-s001.zip › ijms-3266410-supplementary.pdf]
